# Volatile Organic Compounds Emitted by Fungal Associates of Conifer Bark Beetles and their Potential in Bark Beetle Control

**DOI:** 10.1007/s10886-016-0768-x

**Published:** 2016-09-29

**Authors:** Dineshkumar Kandasamy, Jonathan Gershenzon, Almuth Hammerbacher

**Affiliations:** 1 Department of Biochemistry, Max Planck Institute for Chemical Ecology, Hans-Knöll Str. 8, 07745 Jena, Germany; 2 Department of Microbiology, Forestry and Agricultural Biotechnology Institute, University of Pretoria, Private Bag X20, Hatfield, Pretoria, 0028 South Africa

**Keywords:** Symbiosis, Pest management, Fusel alcohol, Aliphatic alcohol, Aromatic compound, Terpenoid, *Ophiostoma*, *Grosmannia*, *Endoconidiophora*, *Ips*, *Dendroctonus*

## Abstract

Conifer bark beetles attack and kill mature spruce and pine trees, especially during hot and dry conditions. These beetles are closely associated with ophiostomatoid fungi of the Ascomycetes, including the genera *Ophiostoma*, *Grosmannia,* and *Endoconidiophora,* which enhance beetle success by improving nutrition and modifying their substrate, but also have negative impacts on beetles by attracting predators and parasites. A survey of the literature and our own data revealed that ophiostomatoid fungi emit a variety of volatile organic compounds under laboratory conditions including fusel alcohols, terpenoids, aromatic compounds, and aliphatic alcohols. Many of these compounds already have been shown to elicit behavioral responses from bark beetles, functioning as attractants or repellents, often as synergists to compounds currently used in bark beetle control. Thus, these compounds could serve as valuable new agents for bark beetle management. However, bark beetle associations with fungi are very complex. Beetle behavior varies with the species of fungus, the stage of the beetle life cycle, the host tree quality, and probably with changes in the emission rate of fungal volatiles. Additional research on bark beetles and their symbiotic associates is necessary before the basic significance of ophiostomatoid fungal volatiles can be understood and their applied potential realized.

**Electronic supplementary material:**

The online version of this article (doi:10.1007/s10886-016-0768-x) contains supplementary material, which is available to authorized users.

## Introduction

Conifer bark beetles are phloem-feeding insects with immense ecological importance in coniferous forest ecosystems throughout the world. By attacking old and wind-thrown trees, these insects serve to rejuvenate forests by recycling nutrients. However, once beetle populations reach a threshold density a number of the more aggressive species attack healthy trees (Bentz et al. 2010; Raffa et al. 2008; Wermelinger 2004). During such outbreaks, bark beetles can destroy millions of hectares of living forest, with great economic and ecological implications. During the last 25 years, bark beetle outbreaks have increased rapidly worldwide as a result of climate change, with the increased frequency of high temperatures, droughts, and windstorms (Kausrud et al. 2012; Kurz et al. 2008). These conditions have allowed bark beetle populations to increase, and particularly to expand their ranges into forest ecosystems containing tree species that are susceptible to attack because of their lack of any prior evolutionary experience with these insects (Bentz et al. 2010; Cudmore et al. 2010; Erbilgin et al. 2014). Hence, there is a strong need for new approaches to bark beetle control.

Aggressive bark beetle species colonize mainly pine and spruce trees via a characteristic sequence of behaviors. First, a pioneer beetle (male or female depending on the species) identifies a suitable host tree and releases aggregation pheromones that attract conspecifics of one or both sexes. The aggregation pheromones are produced *de novo* by the bark beetles, and also by utilizing some of the host compounds as precursors. Additionally, some microbes associated with bark beetles may play a role in pheromone production (Blomquist et al. 2010; Brand et al. 1976; Vite et al. 1972; Wood 1982a; Zhao et al. 2015). After mating, female beetles construct vertical galleries in the phloem tissue. Once enough beetles are recruited to the host tree, beetles produce short-range anti-aggregation pheromones that repel and divert newly arriving beetles to neighboring trees, thus avoiding intra-specific competition for resources and space. Eggs are laid on the sides of the vertical maternal galleries made by the parent beetles, and the newly hatched larvae make their own feeding tunnels at right angles. Larvae feed on both the phloem tissue and the symbiotic microbes with which they are associated (Ayres et al. 2000; Hodges et al. 1968; Six 2012, 2013). At the end of each larval tunnel, a chamber is excavated where the larva pupates. Adult beetles emerge from the gallery through exit holes and attack new trees under favorable conditions, or overwinter underneath the host tree bark or in the soil (Sauvard 2004). Volatile organic compounds (VOCs) play a role in many stages of the bark beetle life cycle including attraction to hosts, aggregation for mass attack, and repulsion of competitors. The identification of volatile chemicals that act as pheromones and related attractants were landmark achievements in the developing science of chemical ecology (e.g., Silverstein et al. 1966, 1968; Wood et al. 1967). Since then, these compounds have been employed with varying success to trap and monitor bark beetle populations (Bakke 1991), and also could play a role in new control efforts.

The interactions of bark beetles with their conifer hosts have long been known to be mediated at least in part by microbes (Adams et al. 2013; Brand et al. 1976, 1977; Six 2013; Therrien et al. 2015). This concept has expanded in recent years due to our increasing knowledge about insect-microbe symbiosis in general, and how the formation of mutualisms with microbes gives insects access to new resources, supplements their nutrition, and allows them to adapt to niches that are otherwise unfavorable (Janson et al. 2008). Bark beetles have symbiotic associations with fungi including Ascomycetes of the genera *Ophiostoma*, *Grosmannia, Ceratocystiopsis,* and *Endoconidiophora,* and a few species also have associations with Basidiomycetes of the genus *Entomocorticium* (de Beer et al. 2014; Zipfel et al. 2006). *Ophiostoma*, *Grosmannia,* and *Ceratocystiopsis* form a monophyletic group in the Ophiostomatales, whereas *Endoconidiophora* is in the order Microascales (Spatafora and Blackwell 1994). Some of these fungi grow from the beetle galleries into the phloem and sapwood (living xylem) where their dark mycelium causes extensive bluish-grey or blackish discoloration of the wood. The fungi associated with conifer- feeding beetles are saprophytic, such as *Ophiostoma* species, or necrotrophic, such as *Grosmannia* (whose asexual phase is known as *Leptographium*) and *Endoconidiophora* species (de Beer et al. 2014; Harrington 2005). *Ophiostoma* and *Grosmannia* species exhibit either no, weak, or moderate pathogenicity. *Endoconidiophora* species, on the other hand, are highly pathogenic and can kill healthy conifers when artificially inoculated (Krokene and Solhheim 2002). However, the role of *E. polonica* in killing trees is much debated and has not been demonstrated outside of artificial inoculations (Six and Wingfield 2011). The roles of these ophiostomatoid fungi vary greatly depending upon beetle life strategy and species. While the beetle serves as a fungal vector, boring the entry hole and inoculating the host tree, the fungus may supply the beetle with nutrients, degrade host defenses, and help kill the tree, which often is correlated to brood fitness (Bentz and Six 2006; Hammerbacher et al. 2013; Krokene and Solhheim 1998). The close interaction between bark beetle and fungus are likely to be mediated by VOCs emanating from the fungus that attract or repel beetles depending on the species, environmental conditions, or stage of the life cycle. Thus, it may be possible to exploit these compounds for control of bark beetles.

This review explores the possibility that fungal volatiles could be applied to the management of bark beetle outbreaks. We begin by briefly outlining our current understanding of the interactions between frequently studied conifer bark beetles and their symbiotic fungi. Next, we survey the types of chemical compounds emitted by the fungal symbionts and examine what is known about their roles in fungi-beetle relationships. Finally, we discuss the potential uses of these volatiles in controlling bark beetle attacks.

## Fungal Associates Play Important Roles in Bark Beetle Life History

### The Eurasian Spruce Bark Beetle Forms Inconsistent Associations with Ophiostomatoid Fungi

*Ips typographus* L.*,* the Eurasian bark beetle, is the most aggressive primary bark beetle that attacks Norway spruce trees (*Picea abies* (L.) Karst.) in Europe and northern Asia (Christiansen and Bakke 1988). *Ips typographus* has no specialized integumental structures to transport symbiotic microbes, such as sac-like mycangia lined with secretory glands. Instead, several microbe species are carried in non-glandular pit-like structures on the exoskeleton, on pits and punctures of the head and pronotum, on the elytra, and perhaps in the beetle’s gut (Furniss et al. 1990). Pathogenic fungal associates are thought to play an important role in host colonization by *I. typographus* and in accelerating tree death (Krokene and Solhheim 1998). Fungi also may provide nutrients to *I. typographus*, metabolize host toxins, or exhaust tree defenses by over-stimulating the production of oleoresins and phenolic compounds (Hammerbacher et al. 2013; Zhao et al. 2011), but these benefits have not yet been rigorously proven.

Details of the association of *I. typographus* with specific fungi are not well understood because different associates have been isolated from attacked trees in different geographic areas (Table 1), and the composition of fungi changes in bark beetle galleries during different stages of attack. On the geographical level, *Endoconidiophora polonica* (Siemaszko) Z.W. de Beer, T.A. Duong & M.J. Wingf. is reported to be the dominant fungal associate in Norway, Poland, and Austria (Kirisits 2010; Krokene and Solhheim 1996). However, in other regions, *Ophiostoma bicolor* R.W. Davidson & D.E. Wells or *Grosmannia penicillata* (Grosmann) Goid. are reported to be predominant (Linnakoski et al. 2016; Persson et al. 2009; Repe et al. 2013; Viiri and Lieutier 2004). Other ophiostomatoid fungi that have been found in association with *I. typographus* in several regions include *Grosmannia europhioides* (E.F. Wright & Cain) Zipfel, Z.W. de Beer & M.J. Wingf.*, Ophiostoma ainoae* H. Solheim and *Ophiostoma piceae* (Münch) Syd & P. Syd. (Jankowiak et al. 2009; Kirisits 2010; Persson et al. 2009; Repe et al. 2013; Viiri and Lieutier 2004; Yamaoka et al. 1997). The regional distributions of different ophiostomatoid fungi might be due to differences in temperature optima for growth as well as local adaptations to climate, host chemistry, phloem moisture levels, and even investigation methods (Giordano et al. 2013; Linnakoski et al. 2016; Six and Bentz 2007; Solheim 1991b). To clarify the roles of each fungal species in the interaction with *I. typographus*, comparative studies are needed to determine their abilities to concentrate vital nutrients and detoxify host defense compounds.

**Table 1:** Bark beetles common in spruce and pine forests in Europe and North America and their associated fungal symbiont

Bark beetle	Host tree	Distribution	Associated fungi	Beetle transport site	Division and order	Relationship
*Ips typographus* Eurasian bark beetle	Mainly Norway spruce *(Picea abies)*	Eurasia	*Endoconidiophora polonica*	Exoskeleton, gut	*Ascomycota, Microascales*	-
			*Grosmannia europhioides, Grosmannia penicillata, Ophiostoma bicolor, Ophiostoma ainoae, Ophiostoma piceae*	Exoskeleton, gut	*Ascomycota, Ophiostomales*	-
*Dendroctonus ponderosae* Mountain pine beetle	All pine trees	North America	*Grosmannia clavigera*	Mycangia, exoskeleton	*Ascomycota, Ophiostomales*	Obligate mutualist
			*Ophiostoma montium*	Mycangia, exoskeleton		Obligate mutualist
			*Leptographium longiclavatum*	Mycangia, exoskeleton		Mutualist
*Dendroctonus frontalis* Southern pine beetle	All pine trees	Southern United States	*Entomocorticium* sp. A.	Mycangia	*Basidiomycota, Russulales*	Obligate mutualist
			*Ceratocystiopsis ranaculosus*	Mycangia	*Ascomycota, Ophiostomales*	Mutualist
			*Ophiostoma minus*	Exoskeleton	*Ascomycota, Ophiostomales*	Antagonist
*Ips pini* Pine engraver beetle	Mostly weak and dead pine trees	North America	*Ophiostoma ips*	Exoskeleton	*Ascomycota, Ophiostomales*	Conditional mutualist
*Dendroctonus rufipennis* North American spruce beetle	All spruce species	The Rocky Mountains (North America)	*Leptographium abietinum*	Exoskeleton	*Ascomycota, Ophiostomales*	Conditional mutualist

In the spruce forests of southern Norway and northeastern Poland, *E. polonica* is frequently the first ophiostomatoid fungus that establishes in phloem tissues adjacent to the parent gallery because it can grow well under low oxygen and high moisture conditions and thus has a competitive advantage as the primary invader in fresh wood (Kirisits 2010; Solheim 1992). *Endoconidiophora polonica* also can detoxify host chemical defenses that are induced upon beetle attack, thereby providing an additional benefit to beetle larvae and adults (Wadke et al. 2016). Once *I. typographus* is established in the tree and *E. polonica* grows into the sapwood, other fungi such as *O. bicolor*, *G. penicillata,* and *G. europhioides* may successively appear in the phloem around the larval galleries (Solheim 1991a, 1992). For example, *G. penicillata* was shown to be better adapted to grow in the phloem tissues than in sapwood, and often forms dense lawns of asexual spores in pupal chambers. *Endoconidiophora polonica,* on the other hand, was reported to occur less frequently during the pupal and adult stages of the beetle (Kirisits 2004).

### The North American Spruce Beetle Has a Consistent Fungal Partner

The North American spruce beetle, *Dendroctonus rufipennis* Kirby, occurs throughout all spruce (*Picea spp*.) habitats in North America, and has caused severe forest mortality in the Rocky Mountains in recent years (Hart et al. 2014; Maroja et al. 2007). Like the Eurasian spruce bark beetle, *D. rufipennis* does not possess glandular mycangia. Despite this, *D. rufipennis* frequently is associated with *Leptographium abietinum* (Peck) M.J. Wingf. throughout its range (Six and Bentz 2003) (Table 1). Studies of the fungal populations associated with *D. rufipennis* in nature have shown an 80–100 % incidence of *L. abietinum* (Aukema et al. 2005; Six and Bentz 2003). Although the *in vivo* benefit of *L. abietinum* to *D. rufipennis* is not completely understood, this fungus produces ergosterol (Bentz and Six 2006), a steroid which is required by many insect species to produce hormones for various developmental processes (Clayton 1964; Mondy and Corio-Costet 2000), However, plants often contain sterols only in low amounts or in forms inaccessible to insects. Therefore, bark beetles that feed on *L. abietinum* and other fungi may be able to supplement their diet with essential sterols. A recent study showed that *D. rufipennis* feeding under artificial conditions on *L. abietinum* gained considerably more weight and had higher survival rates compared to beetles feeding on the same diet without *L. abietinum*. However, the study also showed that the presence of *L. abietinum* negatively affected oviposition and gallery construction by *D. rufipennis in vitro* (Cardoza et al. 2008). It was, therefore, concluded that the association with *L. abietinum* may provide nutritional benefits to the beetle, but might also have antagonistic effects.

### Pine-Infesting Bark Beetles often Form Symbiotic Relationships with Specific Fungi

*Dendroctonus ponderosae* Hopkins, the mountain pine beetle, is the most well-studied species of all the conifer colonizing bark beetles. It is indigenous to western North America and primarily attacks lodgepole pine and other pine species (Wood 1982b). *Dendroctonus ponderosae* mainly vectors fungi such as *Grosmannia clavigera* (Robinson-Jeffrey & R.W. Davidson) Zipfel, Z.W. de Beer & M.J. Wingf., *Ophiostoma montium* (Rumbold) Arx and *Leptographium longiclavatum* S.W. Lee, J.J. Kim & C. Breuil (Table 1) (Six 2012). While *G. clavigera* is carried predominantly in sac-like mycangia located on the maxillary cardines (a portion of the mouthparts) as well as on the exoskeleton, *O. montium* often is seen in larger numbers on the exoskeleton than in mycangia (Six 2003; Whitney and Farris 1970). The mycangial secretions support the yeast-like cell division of spores, providing a continuous supply of inoculum to *D. ponderosae* for an extended period of time during host colonization (Bleiker et al. 2009).

With its long co-evolutionary history with *D. ponderosae*, *G. clavigera* is reported to be much more aggressive during host colonization compared to *O. montium,* which is only moderately pathogenic to pine trees (Solheim and Krokene 1998). *Grosmannia clavigera* tolerates the high levels of monoterpenes in freshly attacked bark by employing specific ATP-binding cassette transporters (ABC) that export monoterpenes from the fungal cell (Wang et al. 2013). This species also can utilize monoterpenes as a carbon source, making it exceptionally well-adapted for survival in resinous bark and wood tissue (Wang et al. 2014)*.*

*Dendroctonus ponderosae* and its fungi exhibit a mutualistic symbiosis. Both *G. clavigera* and *O. montium* play an essential role in *D. ponderosae* development by concentrating nitrogen and producing ergosterol, which is critical for beetle development and reproduction (Bentz and Six 2006). *Grosmannia clavigera* concentrates nitrogen better than *O. montium,* possibly by assimilating it from sapwood and transporting it to the phloem tissues (Bleiker and Six 2007; Cook et al. 2010). Beetles that fed on phloem colonized by *G. clavigera* emerged faster and produced more offspring with larger body sizes than beetles fed on *O. montium,* which in turn were larger than beetles reared without fungi (Bleiker and Six 2007; Six and Paine 1998). This shows that two mutualist fungi of *D. ponderosae* differ in the scale of the benefits they offer to the beetle and of the two, *G. clavigera* can be considered superior. Despite this observation, the developing larvae preferentially fed on phloem infested with both fungi over phloem infested with either species alone, indicating complementary benefits (Bleiker and Six 2007). It appears that feeding on spores by newly eclosed, sexually immature (teneral) adult beetles is necessary for reproduction. Teneral adults that fed on spores of mutualistic fungi produced in the pupal chamber consumed little phloem before emerging. In contrast, when spores were not produced in the pupal chamber, teneral adults tunneled extensively into the phloem tissues (Bleiker and Six 2007). Newly emerged adults that failed to feed on spores produced few egg galleries and laid no eggs (Six and Paine 1998).

Temperature plays a major role in determining the relative proportions of the two fungi in a given population of *D. ponderosae*. *Grosmannia clavigera* dominates at cooler locations with temperature optima around 20 °C, whereas *O. montium* dominates in warmer areas with optimum growth close to 30 °C (Moore and Six 2015; Six and Bentz 2007). However, during *in vitro* competition, *G. clavigera* captures more resources at most temperatures compared to *O. montium* (Moore and Six 2015). Interestingly, sporulation of *G. clavigera* peaked at 30 °C, which is suboptimal for the growth of this fungus, whereas *O. montium* sporulated at low levels across all temperatures. As global average temperature is predicted to rise in coming years, a temperature-driven model predicted that in a few decades, *O. montium* may come to dominate this symbiosis (Addison et al. 2013). These studies collectively showed that temperature can differentially affect growth, resource capture, and sporulation of the two mutualistic symbionts in space and time, which in turn could influence the population dynamics of *D. ponderosae*.

The southern pine beetle, *Dendroctonus frontalis* Zimmermann*,* is the most destructive bark beetle species within its natural range in the southern United States, attacking healthy pine trees when population levels are high (Ungerer et al. 1999). With its rapid generation time and fast dispersal rate, *D. frontalis* is an economically important pest that causes especially severe damage in regions affected by drought and high temperatures. It has been suggested that a rise of 3 °C in minimum temperature would allow the *D. frontalis* population to move northwards and expand its natural range to naïve pine forests with no prior evolutionary exposure to this threat (Ungerer et al. 1999). The reproductive success of *D. frontalis* depends mainly on two mutualist fungi, the basidiomycete *Entomocorticium* sp. A and the non-staining ascomycete *Ceratocystiopsis ranaculosus* J.R. Bridges & T.J. Perry*,* which are carried in the prothoracic mycangia of female beetles (Barras and Perry 1972.; Happ et al. 1971) (Table 1). The developing larvae receive their nutrition by feeding on *Entomocorticium* sp. A. and *C. ranaculosum* growing within and adjacent to the feeding tunnels (Barras 1973; Bridges and Perry 1985). The basidiomycete, *Entomocorticium* sp. A is more beneficial to the bark beetle than the other associate, *C. ranaculosum,* in terms of both total nitrogen content in the hyphae and in concentrating nitrogen in the phloem. *Dendroctonus frontalis* that develop together with *Entomocorticium* sp. A also are larger, with higher lipid content and higher fertility than those that develop with the other associate, *C. ranaculosum* (Ayres et al. 2000; Coppedge et al. 1995; Goldhammer et al. 1990).

A close relative of *D. frontalis, Dendroctonus brevicomis* LeConte (the western pine beetle), also possesses a similar set of mutualistic symbionts - *Entomocorticium* sp. B, a basidiomycete and *Ceratocystiopsis brevicomis* Hsiau & T.C. Harr.*,* an ascomycete (Bracewell and Six 2014). An experiment to evaluate the dependence and fidelity of *D. brevicomis* towards its symbiotic fungi showed that *Entomocorticium* sp. B is crucial for successful development of this beetle because beetles reared without this fungus produced no offspring. Furthermore, this experiment showed that beetle fitness did not vary when grown together with natal (isolated from same beetle population used in the study) and non-natal (genetically distinct isolate from geographically distinct beetle population) fungal isolates. Interestingly, emerging adults incorporated only the natal isolate into the mycangium and avoided the non-natal isolate (Bracewell and Six 2015).

Not all ophiostomatoid fungi are mutualists or commensals of their associated beetles. For example, the blue-stain fungus *Ophiostoma minus* (Hedgcock) Syd. & P. Syd., which is often encountered in larval galleries of *D. frontalis*, is a strong antagonist to this beetle. *Ophiostoma minus* is a mutualist of phoretic mites (commensal organisms which use beetles as a means of transport) that feed on this fungus and reproduce faster in its presence. Beetle larvae that fed on the portion of the phloem colonized by *O. minus* avoided this fungus by making long tunnels but eventually died (Barras 1970; Hofstetter et al. 2006a). The exact mechanism of antagonism is not known, but production of bioactive polyphenols by *O. minus* may explain avoidance by *D. frontalis.* (Hemingway et al. 1977). *Ophiostoma minus* also was reported to grow faster than the two mutualistic Southern pine beetle fungi by capturing more resources, which was shown to have a strong influence on beetle population dynamics (Hofstetter et al. 2006a, b).

The pine engraver beetle, *Ips pini* (Say) is a native species widely distributed in North America which preferentially attacks stressed, wind-blown, and dead mature pine trees of all species within its geographic range. *Ophiostoma ips* (Rumbold) Nannf., the most dominant fungal associate of this species, is carried in pit-like mycangia on the exoskeleton of this bark beetle (Furniss et al. 1995) (Table 1). This fungus is a generalist, sap-staining pathogenic fungal associate of other conifer-infesting bark beetles as well, occurring in many parts of the world (Suh et al. 2013 *;*Zhou et al. 2002). *Ophiostoma ips* has both positive and negative effects on *I. pini,* and the effects vary based on the timing of fungal establishment. For example, when *O. ips* was introduced in logs before the beetle, there was a reduction in the entry of females, but the presence of *O. ips* in larval galleries increased brood emergence. However, when both fungus and beetle were introduced at the same time, there was no noticeable difference in brood development and adult emergence (Kopper et al. 2004; Yearian et al. 1972). This study indicated that *I. pini* might use volatile cues arising from its symbiotic fungus to evaluate the extent of host colonization by conspecific beetles, thus avoiding crowding (Kopper et al. 2004).

## Volatiles from ophiostomatoid Fungi and their Effects on Bark Beetles

Fungi often emit complex mixtures of various low molecular weight compounds with a distinctive odor. These volatile organic compounds have been studied for many years in the food and flavor industries, and even serve as biomarkers for identification of harmful molds in agriculture and fungal infestations in buildings. The volatile blend produced by a fungus varies with respect to growth conditions such as temperature, substrate, and time. Additionally, different genotypes within the same or sympatric species exhibit both qualitative and quantitative differences in their profiles of volatiles (Mburu et al. 2013; Weikl et al. 2016).

Fungal volatiles are known to facilitate many of the associations between fungi and insects, acting as pheromones, kairomones, and allomones. However, the volatiles emitted by the fungal partners of bark beetles have received little study. Thus, we collected volatiles from ten species of beetle-associated, ophiostomatoid fungi grown in potato dextrose broth and searched the literature for the effects of these *de novo* synthesized volatiles on bark beetles. The results are given in Table 2 and described in the following sections. To determine if the volatiles emitted when fungi are grown in potato dextrose broth are similar to those emitted under natural conditions, we compared the emission profiles of several *I. typographus*-associated fungi, including *E. polonica*, *O. bicolor*, *O. piceae*, *G. europhioides,* and *G. penicillata*, grown on potato dextrose broth (Supplemental material) with the emission profiles when grown on spruce bark. There were few qualitative or quantitative differences between these two types of media for most species, indicating that the volatiles we detected from fungi grown on potato dextrose broth are likely to be emitted under natural conditions.

**Table 2:** List of some fungal volatiles identified from ophiostomatoid fungi as detected in our collections. Only selected volatiles are listed emphasizing compounds previously shown to have activity with bark beetles. Fungi were grown in potato dextrose broth, headspace volatiles collected on sorbent, and compounds analyzed by thermal desorption-gas chromatography-mass spectrometry (more information given in supplemental section). Bark beetles reported to respond to these volatiles and the behavioral significance of these volatiles for the beetles are also listed

Fungal volatile	Emitting ophiostomatoid species	Responding bark beetle species	Behavioral response of beetle	References
Fusel alcohols and acetates				
Isoamyl alcohol	*E. polonica, G. clavigera, G. penicillata, G. europhioides, O. bicolor, O. piceae, O. minus, O. ips, L. abietinum*	*D. frontalis*	Synergist of attractant	(Brand et al. 1977)
Isoamyl acetate	*E. polonica, G. penicillata, G. europhioides, O. bicolor, O. piceae, O. minus,*	*D. frontalis*	Synergist of attractant	(Brand et al. 1977)
2-Phenylethanol	*E. polonica, G. clavigera, G. penicillata, G. europhioides, O. bicolor, O. piceae, O. minus, O. ips, L. abietinum*	*D. frontalis*	Anti-aggregant	(Sullivan et al. 2007)
		*D. ponderosae*	Anti-aggregant	(Pureswaran et al. 2000)
		*I. typographus, I. pini*	No response	(Borden et al. 2004; Schlyter et al. 1987)
2-Phenylethyl acetate	*E. polonica, G. penicillata, G. europhioides*	*D. frontalis*	Synergist of attractant	(Brand et al. 1977)
Aliphatic alcohols				
1-Hexanol	*G. europhioides, O. bicolor, O. piceae, O. minus*	*I. typographus, D. ponderosae, D. frontalis*	Synergist of repellent	(Borden et al. 1998; Dickens et al. 1992; Zhang et al. 1999)
		*D. rufipennis*	Anti-aggregant	(Poland et al. 1998)
		*I. pini*	No response	(Huber et al. 2001)
1-Octanol	*O. piceae, O. minus, L. abietinum*	-	-	
1-Nonanol	*O. piceae, O. minus, L. abietinum*	-	-	
Aromatic compounds				
Benzyl alcohol	*G. penicillata, O. piceae, O. ips*	*D. ponderosae, I. pini, D. rufipennis, D. brevicomis*	Synergist of anti-aggregant	(Borden et al. 1998; Huber et al. 2001)
Methyl cinnamate	*O. ips*	-	-	
Ethyl cinnamate	*O. ips*	-	-	
Ethyl benzoate	*O. ips*	-	-	
Acetophenone	*O. ips*	*D. ponderosae, I. pini, D. rufipennis*	No response	(Pureswaran and Borden 2004; Pureswaran et al. 2000)
		*D. brevicomis, D. pseudotsugae, D. frontalis,*	Anti-aggregant	(Erbilgin et al. 2008; Pureswaran and Borden 2004)
2,3-Dihydrobenzofuran	*O. ips*	-	-	
Terpenoids				
Geranyl acetone	*E. polonica, O. bicolor, G. clavigera*	*Ips subelongatus*	Anti-aggregant	(Zhang et al. 2007)
		*Tetropium fuscum* (Cerambycid beetle)	Pheromone precursor	(Mayo et al. 2013)
(*E*)-β-Caryophyllene	*G. penicillata*	*Pityogenes bidentatus*	Synergist of anti-aggregant	(Byers et al. 2004)
		*D. armandi*	Synergist of attractant	(Zhang et al. 2010)

### Fusel Alcohols and their Acetates

Fusel alcohols are low molecular weight aliphatic and aromatic alcohols produced by degradation of amino acids via the Ehrlich pathway (Hazelwood et al. 2008). The Ehrlich pathway involves a transamination step in which the amino group is exchanged for an oxygen, resulting in an α-keto acid. Decarboxylation then forms an aldehyde, which then can be reduced to a fusel alcohol by an alcohol dehydrogenase. These alcohols, derived mainly from phenylalanine, valine, leucine, isoleucine, and methionine, can be further modified by esterification of the alcohols to form acetates with strong odors (Pires et al. 2014) that are extensively utilized in the food and flavor industries.

**Fig. 1:**
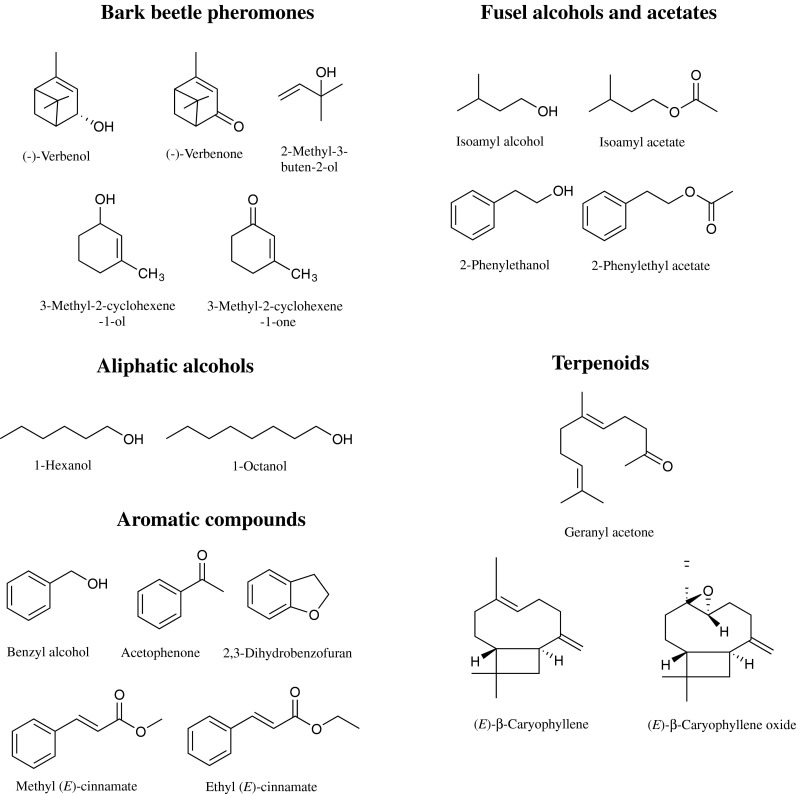
Chemical structures of bark beetle pheromones and major volatiles emitted by ophiostomatoid fungal associates of conifer bark beetles

Fusel alcohols are produced mainly by fungi, and the aromas of these compounds attract many fungivorous insects such as nitilulid beetles and fruit flies, which disperse the emitting microbes to new colonization sites (Bartlet and Wicklow 1999; Christianens et al. 2014). Fusel alcohols (e.g., isoamyl alcohol and 2-phenylethanol, Fig. 1) and their acetate esters (isoamyl acetate and 2-phenylethyl acetate), were produced by several species of *Ophiostoma, Ceratocystis, Grosmannia,* and *Endoconidiophora* in our collections (Table 2) with the rate of emission varying among species. These compounds may play a role in bark beetle-ophiostomatoid fungus associations by attracting beetles to their symbionts, or to symbiont habitats. Although fungal symbionts are expected to be present already in beetle galleries during initial attack on the host tree, volatiles may keep beetle feeding closely synchronized with areas of fungal growth, thus allowing beetles to maximize the benefits from this association. In theory, fungal volatiles could also repel beetles by signaling host trees or areas of host trees that are already under attack by competing beetles. However, published data mostly report attraction of beetles to specific fungal volatiles rather than repulsion. For example, isoamyl acetate and 2-phenylethyl acetate have been reported to attract *D. frontalis* in laboratory assays when added to unattractive concentrations of pheromone blends, either separately or together (Brand et al. 1977). Additionally, isoamyl alcohol and 2-phenylethanol together with their acetates efficiently synergized the attractiveness of bait mixtures used to capture *D. frontalis* (Brand et al. 1977). On the other hand, 2-phenylethanol was shown to be a strong anti-aggregation component for *D. ponderosae* and *D. frontalis* when added to their pheromone blends (Pureswaran et al. 2000; Sullivan et al. 2007). 2-Phenylethanol also was found in hindgut extracts of *I. typographus* and *I. pini*, and elicited strong antennal responses from both sexes, but addition of 2-phenylethanol to the respective pheromone blends of these beetle species did not significantly alter attraction (Borden et al. 1998; Pureswaran et al. 2000; Schlyter et al. 1987). The role of fusel alcohols in the attraction of other bark beetle species is unknown.

### Aliphatic Alcohols

Aliphatic alcohols are produced via the oxidation and cleavage of polyunsaturated fatty acids such as linoleic acid. Although the exact pathways in fungi still need to be elucidated, it is known that biosynthesis first involves oxidation of fatty acids by enzymes such as lipoxygenases or fatty acid diol synthases. The resultant hydroperoxide intermediates are cleaved by hydroperoxide lyases using a hemolytic cleavage mechanism to form short- or medium-chain aliphatic alcohols (Combet et al. 2006).

### 1-Hexanol

Bark beetles have been shown to reject non-host tree species due to the absence of host cues or the presence of non-host volatiles. 1-Hexanol (Fig. 1) is a green leaf volatile (GLV), one of a group of C_6_ alcohols, aldehydes, and esters emitted by the foliage of many angiosperms. We found that ophiostomatoid fungi such as *G. europhioides, O. bicolor, O. piceae,* and *O. minus* also produce 1-hexanol when cultivated in the laboratory (Table 2). The compound has been described to be a synergistic repellent (a compound that repels only in combination with other compounds, but not by itself) for *I. typographus, D. ponderosae,* and *D. frontalis* (Borden et al. 1998; Dickens et al. 1992; Zhang et al. 1999). Additionally, 1-hexanol is the only known GLV that disrupts *D. rufipennis* attraction to its pheromone blend (Poland et al. 1998). *Ips pini*, on the other hand, showed no behavioral response to 1-hexanol and other GLVs, although the GLVs hexanal and (*E*)-2-hexenal elicited strong antennal responses from this species (Huber et al. 2001).

### 1-Octanol and 1-Nonanol

*Ophiostoma piceae, O. minus,* and *L. abietinum* emitted 1-octanol and 1-nonanol in our collections (Fig. 1, Table 2). While it is not known whether these compounds elicit electrophysiological and/or behavioral responses from conifer-infesting bark beetles, aliphatic alcohols are reported to attract insect parasitic nematodes (Nematoda: Rhabditidae) that feed on bark beetle-associated microbes (O’Halloran and Burnell 2003). In addition, a number of nematode species have been cultured on ophiostomatoid fungi. For example, some species of nematodes associated with *D. rufipennis* were successfully cultured and maintained on *L. abietinum* (Cardoza et al. 2008), while a parasitic nematode, *Parasitorhabditis* sp*.*, isolated from different body parts of the red turpentine beetle, *Dendroctonus valens,* was maintained and reproduced on sporulating cultures of *O. minus* (Hunt and Poinar 1971). Similarly, *O. minus* was shown to support the growth of the pathogenic pinewood nematode, *Bursaphelenchus xylophilus* (Steiner & Buhrer) Nickle (Maehara and Futai 1997)*.* It is not yet known if fungi such as *O. minus* and *L. abietinum* actually attract phoretic nematode species (those carried by other organisms) in nature, if fungi are vectored by nematodes, and what effect phoretic nematodes have on bark beetles, but fungal volatiles could conceivably play a role in these interactions.

### Sesquiterpenes

Sesquiterpenes are produced by fungi from the mevalonate pathway intermediates dimethylallyl diphosphate and isopentenyl diphosphate, which are condensed to produce the C_15_ farnesyl diphosphate. This linear polyprenyl diphosphate then is further transformed to linear and cyclic products by terpene synthase enzymes. Further modifications of these products via oxidation reactions mediated by cytochrome P450 enzymes are common in nature, but such reactions have not yet been described in fungi, only in other microbes and higher plants (Keller et al. 2005).

### Geranylacetone

Produced by the oxidation of the sesquiterpene alcohols farnesol or nerolidol (Mayo et al. 2013), geranylacetone (Fig. 1) was detected from three blue stain fungi, *E. polonica, G. clavigera,* and *O. bicolor* in our collections (Table 2). The Asian larch bark beetle, *Ips subelongatus* Motschulsky, demonstrated strong antennal responses to geranylacetone, and this compound significantly disrupted the attractiveness of pheromone mixtures in field experiments (Zhang et al. 2007). Antennae of *I. typographus* also have been reported to respond to geranylacetone, but it is not known whether this compound mediates the behavior and ecology of *I. typographus* and most other bark beetle species. However, cerambycid beetles such as the brown spruce longhorn beetle, *Tetropium fuscum* (Fabricius)*,* and the eastern larch borer, *T. cinnamopterum* Kirby*,* utilize geranylacetone as a precursor to their aggregation pheromone, fuscumol, the alcohol analog of geranylacetone (Mayo et al. 2013). Ophiostomatoid fungi producing this compound might, therefore, be attractive to these secondary beetles.

### (*E*)-β-Caryophyllene

The bicyclic sesquiterpene, (−)-(*E*)-β-caryophyllene (= (−)-β-caryophyllene, Fig. 1) is produced by several fungi mostly in their later growth phases (Kramer and Abraham 2012). The generalist ophiostomatoid fungus, *G. penicillata,* emitted this compound and its epoxide, caryophyllene oxide, in our volatile collections (Table 2). The significance of (*E*)-β-caryophyllene and caryophyllene oxide in fungi is not well understood, but these compounds have been reported to have both repellent and anti-fungal properties (Boulogne et al. 2012). For example, the leaves of the legume *Hymenaea courbaril* L. in Costa Rica, which is seldom foraged on by the leaf-cutting ant *Atta cephalotes* L.*,* contain both (*E*)-β-caryophyllene and caryophyllene oxide. Performance assays indicated that leaf cutting ants avoided caryophyllene oxide more than (*E*)-β-caryophyllene, and preferred plants became repellent when treated with these compounds. Additionally, these compounds were reported to have anti-fungal activity against the obligate mutualist fungus farmed by the ants in their nests (Hubbell et al. 1983). Given the repellency and anti-fungal activity of these sesquiterpenes, ophiostomatoid fungi that produce these compounds may be repellent to beetles or able to inhibit the growth of competing microbes. For example, (*E*)-β-caryophyllene was reported to disrupt the attraction of *Pityogenes bidentatus* (Herbst), a small bark beetle that colonizes diseased or weakened branches of Scots pine, *Pinus sylvestris* L.*,* to its aggregation pheromone (Byres et al. 2004). On the other hand, increased numbers of *Dendroctonus armandi* Tsai & Li, a serious pest of Chinese white pine, *Pinus armandii* Franch., were captured when (*E*)-β-caryophyllene was present in combination with other host semiochemicals such as α-pinene (Zhang et al. 2010). Other research on (*E*)-β-caryophyllene showed that when this compound was produced by certain maize lines, entomopathogenic nematodes that prey on maize root herbivores, such as *Heterorhabditis megidis* Poinar, Jackson & Klein*,* were recruited (Rasmann et al. 2005). The recruitment of nematodes by (*E*)-β-caryophyllene-producing ophiostomatoid fungi may have important consequences for their associated bark beetle species.

### Aromatic Compounds

Although little is known about the biosynthesis of volatile aromatic compounds in fungi, carbon-labelling studies showed that the formation of phenylpropanoid-derived substances is similar to that in plants proceeding from the amino acid L-phenylalanine, itself a product of the shikimate pathway (Lapadatescu et al. 2000). In the first step, phenylalanine is deaminated to form cinnamic acid, which forms the basic backbone of all aromatic VOCs. This structure can be further modified by methylation, esterification, chain shortening, reduction, oxidation, or chain cyclization (Widhalm and Dudareva 2015). Other aromatic compounds are derived from the polyketide pathway or other intermediates of amino metabolism and the shikimate pathway.

### Benzyl Alcohol

*Grosmannia penicillata, O. piceae,* and *O. ips* emitted benzyl alcohol in our collections (Fig. 1) (Table 2), a compound that also is found in some non-host angiosperms, such as *Populus tremula* L*.* Benzyl alcohol was reported to elicit antennal responses from *D. ponderosae, I. pini, D. rufipennis,* and *D. brevicomis* (Huber et al. 2000). Benzyl alcohol is a non-host volatile that disrupts aggregation of *D. ponderosae* in ternary and quaternary blends with GLVs and other non-host compounds (Borden et al. 1998). Similar behavioral activity was observed in *I. pini* and the Douglas-fir beetle, *Dendroctonus pseudotsugae* Hopkins (Huber et al. 2001). *Ips typographus*, however, displayed no antennal or behavioral response to benzyl alcohol (Zhang et al. 2000).

### Acetophenone

The aromatic ketone acetophenone (Fig. 1) was emitted only by *Ophiostoma ips* in our collections, and has not been reported previously from any ophiostomatoid fungus. However, this compound has been detected in several species of bark beetles. Acetophenone was identified in hindgut extracts or odors of *D. ponderosae*, *D. rufipennis*, *D. pseudotsugae*, *I. pini*, *D. frontalis,* and *D. brevicomis* (Erbilgin et al. 2007; Pureswaran and Borden 2004; Pureswaran et al. 2000; Sullivan 2005). It has been reported to be a strong anti-aggregant for the western pine beetle, *D. brevicomis,* and even is superior to verbenone as a repellent (Erbilgin et al. 2008). Interestingly, acetophenone did not inhibit the attraction of the western pine beetle predator, *Temnochila chlorodia* (Mannerheim) (Coleoptera: Trogositidae), to beetle aggregation pheromones, thus resulting in a high predator to prey ratio in baited traps, whereas verbenone added to this lure inhibited predator attraction to western pine beetle (Erbilgin et al. 2008). Attraction of *D. frontalis* to its pheromone blend also is inhibited by addition of acetophenone, and a similar effect was reported for *D. pseudotsugae* (Pureswaran and Borden 2004). On the other hand, acetophenone showed no behavioral effect on *D. ponderosae, I. pini,* and *D. rufipennis*, but did elicit antennal responses in both sexes of *D. ponderosae* and *I. pini* (Pureswaran et al. 2000). Because the acetophenone-producing *O. ips* is a fungal symbiont of *I. pini*, it may be fitting that this compound has no behavioral effect on *I. pini*, but repels other bark beetles.

### 2, 3-Dihydrobenzofuran

Also known as coumaran, 2,3-dihydrobenzofuran and its derivatives (Fig. 1) have been widely reported as anti-feedants or insecticides for many polyphagous insects (Morimoto et al. 1999b). This compound is a natural fumigant, and the most common secondary metabolite of plants in the family Cyperaceae, which includes many common wetland weeds (Morimoto et al. 1999a). Its insecticidal properties are attributed to its ability to inhibit acetylcholinesterase, which degrades the neurotransmitter acetylcholine. Inhibition of this enzyme results in excessive buildup of the neurotransmitter at the synaptic junctions, which causes prolonged neural excitation and ultimately leads to death (Rajashekar et al. 2014). 2,3-Dihydrobenzofuran occurred in our volatile collections from *O. ips* cultures under laboratory conditions (Table 2); this is the first report of the identification of such a fumigant from a fungus. Its activity as a volatile insecticide and anti-feedant suggests potential for utilization in bark beetle management.

### Methyl and Ethyl (*E*)-Cinnamate

The volatile aromatic esters, methyl and ethyl cinnamate (Fig. 1) may participate in complex interactions among fungi, beetles, host trees, and nematodes. These compounds are known to have strong nematicidal activity even at low concentrations against the pinewood nematode, *B. xylophilus,* the causal agent of pine wilt disease (Kim et al. 2011). Longhorn beetles in the genus *Monochamus,* which are widely distributed across pine forests globally, are important vectors of the pinewood nematode (Akbulut and Stamps 2012). Some longhorn beetle species also vector ophiostomatoid fungi such as *O. minus* and *O. ips* along with the pinewood nematode. These nematodes utilize ophiostomatoid fungi as nutrient sources and the presence of *O. minus* in beetle galleries increases the number of nematodes carried by emerging beetles (Maehara and Futai 1997). However, the nutritional advantage of *O. ips* to the pinewood nematode and its beetle vector is not known. Methyl and ethyl cinnamate were the predominant volatiles emitted from *O. ips* cultures in our collections (Table 2), and the growth of nematicide-producing *O. ips* in pine could be detrimental to pinewood nematodes. Further experiments on the performance of pinewood nematodes in the presence of *O. ips* could give more insights into the role of fungal volatiles in the population dynamics of the nematodes as well as their beetle vectors. In support of the hypothesis that *O. ips* may negatively affect both beetle and nematode populations, the edible fruiting bodies of *Tricholoma matsutake*, which emit high concentrations of methyl cinnamate, deter the mycophagous hexapod, *Proisotoma minuta* (Tullberg) (Collembola: Isotomidae) from feeding on their fruiting bodies (Sawahata et al. 2008).

### Use of Fungal Volatiles by Bark Beetle Predators

Some natural enemies of bark beetles have been shown to locate their prey through volatiles emitted by beetle-associated symbiotic fungi. For example, females of the pteromalid wasps, *Roptrocerus xylophagorum* (Ratzeburg) and *Spathius pallidus* Ashmead, the most common parasitoids of bark beetles in North America, were attracted to volatiles of *O. ips*- and *O. minus*-inoculated pine bolts in olfactometer assays (Sullivan and Berisford 2004). However, in field assays with *O. minus*-inoculated and mock-inoculated bolts, there was no significant difference in attraction to the two treatments. Nevertheless, female *R. xylophagorum* were not attracted to beetle larval or pupal stages alone, but were attracted only to bark colonized by beetles and containing larval feces (Sullivan and Berisford 2004), suggesting a role for fungal associates in providing attractive stimuli. Another study showed that the specialist parasitoids, *Heydenia unica* Cook & Davis and *Rhopalicus pulchripennis* Crawford, which prey on late larval instars of bark beetles, exploit volatiles from *O. ips*, *G. clavigera,* and *O. montium* as attractants, whereas generalist predators and parasitoids mainly use host pheromones and plant volatiles to locate their hosts (Adams and Six 2008; Boone et al. 2008). Other bark beetle parasitoids also may be attracted by the volatiles of beetle-associated fungi. Studies on *I. typographus*-infested Norway spruce bolts showed emission of several oxygenated terpenes and other volatiles that are typically produced by bark beetle-associated microbes or by auto-oxidation of tree resins. A synthetic blend of oxygenated terpenes was found to elicit olfactory responses in chalcid wasps and to be very attractive to these wasps in laboratory bioassays (Pettersson 2001; Pettersson and Boland 2003).

### Oxidation Products of Host Tree Terpenes

The fungal volatile compounds discussed until now are all likely to be synthesized *de novo* by associates of bark beetles since they were detected when fungi were cultured solely on potato dextrose broth. However, fungal volatiles also could be produced by the metabolism of host plant substances. Many plant pathogenic fungi are reported to transform terpene olefins to oxygenated metabolites *in vitro*, such as the well-known horticultural pathogens *Botrytis cinerea* Persoon, *Aspergillus niger* Tieghem, and *Penicillium digitatum* (Persoon) Sacc. Among the ophiostomatoid fungi, *G. clavigera* transforms the host-derived monoterpene limonene to the volatile oxygenated metabolites, carvone, *p*-mentha-2,8-dienol, perillyl alcohol, and isopiperitenol *in vitro* (Wang et al. 2014). This fungus utilizes limonene as a carbon source (DiGuistini et al. 2011; Wang et al. 2013) for growth. Other mountain pine beetle-associated fungi also can use limonene as a carbon source (Wang et al. 2014), and so also may produce volatile oxygenated metabolites of this monoterpene. Because oxygenated monoterpenes have been shown experimentally to attract parasitoids of bark beetles (Pettersson 2001), fungal transformation products of limonene may function as kairomones for natural enemies, and thus negatively impact bark beetle fitness.

The fungal transformation products that have received the greatest attention are those identical to the pheromones produced by bark beetles. For example, a number of yeast species found in *I. typographus* guts or on their exoskeleton, e.g., *Hansenula* and *Candida* spp., convert the aggregation pheromone components (*−*)-*cis*-verbenol and (*−*)-*trans*-verbenol to (−)-verbenone (Fig. 1). The oxidized monoterpene (−)-verbenone inhibits beetle aggregation (Leufven et al. 1984). The precursor verbenols are themselves transformation products, being made from host tree monoterpenes by beetles (Hughes 1973). Similarly, *C. ranaculosum,* the mycangial fungal associate of the southern pine beetle *D. frontalis*, has been shown to convert (−)-*trans*-verbenol to (−)-verbenone and 3-methyl-2-cyclohexen-l-ol to its corresponding ketone, 3-methyl-2-cyclohexen-l-one (Fig. 1). Both ketones are known anti-aggregation pheromone components of several *Dendroctonus* species (Brand et al. 1976). Recently, two fungal symbionts of *I. typographus, G. europhioides* and *G. penicillata*, were shown to produce the major *I. typographus* aggregation pheromone, 2-methyl-3-buten-2-ol (Fig. 1) (Zhao et al. 2015). These examples highlight the important roles that fungal metabolites could play in bark beetle ecology.

## Ophiostomatoid Fungal Volatiles in the Management of Bark Beetle Pests

Integrated pest management of aggressive bark beetle species usually aims to reduce attack on healthy trees when attack severity reaches economic threshold levels. Several practices are commonly employed, such as removal of wind-thrown and infested trees and thinning of conifer stands. However, accessibility to remote areas and economic and environmental constraints often hamper quick removal of large numbers of infested trees (Christiansen and Bakke 1988). Usage of insecticides that specifically target beetle species is another straightforward control strategy that can cause severe mortality to broods at the site of treatment (Grosman et al. 2009). However, insecticides can play at most a relatively small role in managing major outbreaks due to the logistics and economics of applying insecticides over large, remote forested areas. Additionally, the choice of insecticides may have serious ecological implications due to non-target effects, and their usage may be limited by local legislation. Trap trees were successfully used in the past to limit attacks by baiting a cut tree or healthy trees with pheromones or pheromones and insecticides in order to divert beetles from attacking healthy trees. However, the trap tree must be removed from the forest in time to avoid the risk of spill-over infestations to nearby non-baited trees which could quickly lead to outbreaks (Hokkanen 1991). Trap trees treated with insecticides are still in use for small scale infestations because these allow enough time for removal without risking further infestation (El-Sayed et al. 2009).

The management of bark beetle populations with pheromones and other semiochemicals is a “green” alternative to the use of synthetic insecticides. Semiochemicals have been extensively employed to monitor beetle population levels and sometimes to trap beetles to keep their population below the threshold at which they attack healthy trees. However, trapping efforts have been limited in scale and area because of logistical and economic limitations. Both attraction (“pull”) and deterrence (“push”) strategies have been used. The pull strategy employs stimuli such as aggregation pheromones, host volatiles, or visual cues that are either presented alone or combined in specific combinations. This method can be useful for mass trapping of bark beetles, monitoring local beetle populations, or screening for the presence and abundance of exotic beetles that are accidentally introduced (Bakke 1991; Borden 1989). The disadvantages of a pull strategy are the potential of spill-over infestation and the accidental trapping of useful natural enemies that are attracted to pheromones of bark beetles. The push strategy makes use of stimuli that are repellent to beetles and deters them from attacking potential host trees, or from mating and oviposition. Deterrent compounds such as the anti-aggregation pheromone, verbenone, and non-host volatiles (1-hexanol, (*Z*)-3-hexen-l-ol, and (*E*)-2-hexen-l-ol, *trans*-conophthorin, 3-octanol, 1-octen-3-ol) have been tested in field experiments to determine their efficiency (Zhang et al. 1999). For example, aerial application of verbenone- releasing flakes and verbenone bubble caps was shown to significantly reduce the attack rate of mountain pine beetle in pine stands (Gillette et al. 2006; Shea et al. 1992). Combined application of verbenone and non-host volatiles in spruce forests in Sweden and Slovakia was shown to act synergistically, inhibiting or delaying the attack of *I. typographus* in the treatment areas (Schiebe et al. 2011). However, the push strategy cannot be used as a stand-alone method due to the fact that the repelled beetle population could be diverted to unprotected adjacent areas; therefore it has to be combined with other methods for long term management.

The combination of push and pull stimuli, commonly referred as the “push-pull strategy” (Cook et al. 2006) also is used to control bark beetles. Pest beetles are deterred by using push stimuli placed within target stands or on their perimeter, and simultaneously attracted by using stimuli attached to baited traps placed outside the target stand. Stimuli can be delivered in a number of ways in this strategy, as described above, and push-pull methods have been successfully tested against the mountain pine beetle, *D. ponderosae* and *Ips paraconfusus* Lanier attacking Torrey pine (*Pinus torreyana*), an endangered species growing in a limited area of California (Gillette et al. 2012; Shea and Neustein 1995). The choice of traps (baited trees or baited traps), plot size, and trap spacing was shown to influence the method’s efficacy (Borden et al. 2006).

### Possible Applications of Fungal Volatiles

Bark beetle infestations also might be managed by exploiting volatiles released by bark beetle-associated fungi. For this review, we screened a number of ophiostomatoid fungal species for volatile metabolites and identified a wide range of organic compounds that might mediate behaviors of bark beetles, and that could be investigated for bark beetle control. However, in order to best utilize these compounds as next-generation semiochemicals, their emission patterns and the behavioral ecology of the beetle species themselves should be studied in more detail.

### Bark Beetle Management

Fungal volatiles might be especially useful in push-pull strategies where they could synergize the effects of other attractant or repellent components. For example, isoamyl acetate and phenylethyl acetate (Table 2) could be used in combination with a pheromone mix for attracting higher numbers of *D. frontalis* (Brand et al. 1977). Conversely, the natural anti-feedant volatiles produced by the generalist fungus *O. ips*, including 2,3-dihydrobenzofuran and various cinnamic acid derivatives (Table 2, Morimoto et al. 1999b), could augment anti-aggregation mixtures for improved efficiency in deterring a wide range of pine-infesting bark beetles. Acetophenone, produced by *O. ips* (Table 2), already has been reported to strongly repel some *Dendroctonus* species (Erbilgin et al. 2008; Pureswaran and Borden 2004). For the Eurasian spruce bark beetle, *I. typographus,* volatiles from its associated fungus *E. polonica*, such as isoamyl alcohol and 2-phenylethanol (Table 2), also might be useful attractants or repellents. Furthermore, the profiles of volatiles produced by *G. penicillata* and *G. europhioides* contain sesquiterpenes like (*E*)-β-caryophyllene (Table 2) that may be insecticidal or support the growth of entomopathogenic nematodes. Finally, the profile of volatiles produced by *L. abietinum* contains aliphatic alcohols (Table 2), which attract predatory nematodes, and could be used for this purpose. It already has been shown that several species of nematodes associated with *D. rufipennis* can be reared on *L. abietinum* cell cultures (Cardoza et al. 2008).

Fungal volatiles released from trees that are already infested by conspecifics may repel newly arriving beetles (Cardoza et al. 2008; Kopper et al. 2004). At the same time, these volatiles, in synergy with oxygenated terpenes, might serve as kairomones for natural enemies. Several oxygenated terpenes, such as camphor, pinocamphone, and terpinen-4-ol have been shown to attract parasitoids of bark beetles (Pettersson 2001; Pettersson and Boland 2003). Dispensers with fungal volatiles and oxygenated monoterpenes might, therefore, be used for repelling beetles from potential attack areas such as drought-stressed stands, while simultaneously attracting bark beetle predators and parasitoids to infested areas.

The diversity of volatile compounds emitted by bark beetle-associated fungi holds great potential for the development of new semiochemical-based control measures for these insects. Further research on the identification of fungal volatiles and their effect on the behavior of bark beetles and their natural enemies will provide knowledge that could be exploited for protection of conifer forests. At present, however, applications of semiochemicals for bark beetle management in natural forests are hampered by the scale at which they would have to be deployed. In small conifer plantations, on the other hand, these techniques may be effective, and logistically and economically possible. Small plantations even could exploit fungal cell cultures as baits in traps for attracting bark beetles. A recent study showed that a large range of insects encompassing seven different orders were significantly attracted to a cell culture of the ubiquitous fungus, *Aureobasidium pullulans* (De Bary) G. Arnaud ex Ciferri, Ribaldi & Corte, in traps (Davis and Landolt 2013). One might question whether the amounts of volatiles emitted from fungal cell cultures are sufficient for attracting bark beetles, but beetles are able to perceive some volatile compounds in nanogram or even picogram doses (Andersson et al. 2009). Another factor to consider is that emission of volatiles from fungal cultures may be variable. In our experiments, we found that fungal volatile emissions differed with nutrient availability, and that emissions decreased once the growth medium was depleted. Furthermore, the ecological consequences of using living fungal cultures in traps are still unknown. There is a risk of introducing pathogens to naïve forests, and local laws likely will not permit the usage of potentially pathogenic fungi in forest ecosystems.

Employing fungal volatiles may circumvent these problems because pure substances with attractive or repellent activity can be deployed in pheromone dispensers in combination with known commercial products for bark beetle control. As an example, lethal laurel wilt disease in red bay and avocado trees is caused by the fungus *Raffaella lauricola*, which is associated with an invasive ambrosia beetle, *Xyleborus glabratus* (Fraedrich et al. 2008). The major volatile metabolites of *R. lauricola* when cultivated on potato dextrose agar are isoamyl alcohol, isoamyl acetate, ethanol, ethyl acetate, and isobutyl alcohol (Kuhns et al. 2014). In field assays, the synthetic blend of these *R. lauricola* volatiles, together with volatiles that mimic the host tree, synergistically increased the attraction of beetles to traps compared to host volatiles alone, underlining the value of using microbial volatiles together with already available attractive mixtures (Hulcr et al. 2011; Kuhns et al. 2014). Because ambrosia beetles are phylogenetically closely related to bark beetles, similar strategies might also prove effective in controlling bark beetles. However, there is a need to first evaluate the efficiency with which bark beetles can be trapped in the field using synthetic blends of fungal volatiles together with other known attractants or repellents.

### Biomarkers

Identification of ophiostomatoid fungi through chemotyping of their volatiles could be a promising application of the volatile secondary metabolites produced by these microorganisms. Volatiles have previously proven helpful in the differentiation and identification of fungi to the species level (Larsen and Frisvad 1995; Polizzi et al. 2012). Furthermore, researchers also have predicted the ecological function of fungal species based on their volatile emission patterns (Muller et al. 2013). This approach could be employed during attack by a bark beetle like *I. typographus*, for example, to gain a precise overview of the abundance and succession of the different fungal associates present given the distinctive volatile profiles they showed in our survey. In light of the different properties of the various fungal associates, such information would help predict the speed and virulence of bark beetle outbreaks when obtained on a landscape scale. It also could help provide evidence for the degree of association between fungus and beetle, which ranges from obligate to facultative (Table 1). Because volatiles can be measured in the field in real-time throughout the season by using technology such as proton-transfer reaction mass spectrometry, monitoring of profiles of volatiles represents an attractive non-invasive alternative to culture-based methods for determining the occurrence of fungal species. Although all fungal species included in the current study are culturable, the presence of one species may inhibit the growth of others in culture, which could lead to a bias in estimating species abundance that would be avoided by volatile identification in the field. In theory, the spectrum of volatiles emitted by a fungal taxon could be altered by growth with another fungus, but in previous studies co-cultivation did not affect the qualitative emission profiles (Weikl et al. 2016).

The systematics of ophiostomatoid fungi is complex, and collections often are misidentified due to morphological similarities within this group. Because differences in the profiles of volatiles produced by different phenotypes reflect changes on the genetic level, chemosystematics based on volatile organic compounds could be useful to taxonomists and ecologists for better identification and classification of fungi in this group. Studies have shown that cryptic species within a species complex differ significantly in their volatile profiles (Ludwiczuk et al. 2013; Wawrzyniak et al. 2014), and so by using such methods it should be possible to differentiate among the cryptic species previously described in the *G. clavigera* complex (Alamouti et al. 2011).

Monitoring of volatiles could be especially useful in preventing introductions of bark beetles and their associated fungi into new areas. Routine analysis of volatiles emitted from wood shipments originating from foreign sources might be an effective screening method to identify low level infestations of new potentially invasive species. Monitoring fungal volatiles in forest ecosystems also might provide a method for early detection of new bark beetle invasions that could lead to timely eradication of new pest species before they become established.

## Conclusions

Research on the chemical ecology of bark beetles conducted over many years has revealed much about how beetles aggregate and choose host trees. However, most workers have focused on the chemical compounds originating from the beetles and their host trees, with little attention given to chemical signals originating from the beetle’s fungal associates. Given the pivotal role of fungi in the success of bark beetle infestation of host trees, a better knowledge of the chemical interactions between beetle and fungus should substantially increase our understanding of bark beetle life history. This information in turn will be of great value in refining existing techniques for management of bark beetle pest species and developing new approaches. Deployment of fungal volatiles as attractants or deterrents, alone or in combination with other types of semiochemicals, could significantly improve our ability to control these destructive forest pests.

## Electronic supplementary material

ESM 1 (DOCX 14 kb)
