# Supplementary material for: Volatile Organic Compounds Emitted by Fungal Associates of Conifer Bark Beetles and their Potential in Bark Beetle Control
Source: J Chem Ecol. 2016 Sep 29;42(9):952–69. doi: 10.1007/s10886-016-0768-x (PMC5101256; doi:10.1007/s10886-016-0768-x)
Supplement: Supplementary file 1 — (DOCX 14 kb) [file 10886_2016_768_MOESM1_ESM.docx]

**METHODS FOR FUNGAL CULTURE, HEADSPACE COLLECTION AND VOLATILE ANALYSIS**

**Fungal Culture**

Fungal species were obtained from the culture collections of the Forestry and Agricultural Biotechnology Institute (University of Pretoria, South Africa), the Norwegian Landscape Institute (Ås, Norway) and the Plant-Pathogen Interactions Group, Department of Biochemistry, at the Max Planck Institute for Chemical Ecology (Jena, Germany). Each species was grown on agar plates for 3-5 d. Using a 4 mm sterile cork borer, three to four agar plugs from each plate were inoculated in a 50 ml Erlenmeyer flask containing 20 ml of potato dextrose broth and incubated at room temperature without shaking.

**Sampling of Head Space Fungal Volatile Compounds *in vitro***

Aliquots of 200 µl of fungal broth were removed periodically from culture flasks and transferred to 1.5 ml screw cap glass vials. For headspace analysis, polydimethylsiloxane (PDMS) sorbent, commercially available as silicone tubes (STs) (5 mm) was prepared (Kallenbach et al. 2014). A single ST was hung in each glass vial containing fungal volatiles with the help of manually crafted metal hook attached to bottom of the screw cap. After two hours of adsorption, STs were transferred into clean 1.5 ml brown glass vials and stored at -20°C before analysis.

**Thermal Desorption-Gas Chromatography-Quadrupole Mass Spectrometry (TD-GC-QMS) Analysis**

Volatile samples were analyzed in a TD-GC-QMS system equipped with a TD-20 thermal desorption unit (Shimadzu), a GC Cryo-Trap (Tenax®), and a GC-2010 plus system connected to a quadrupole GC-MS-QP2010 Ultra (Shimadzu). A single ST was placed inside an 8 mm glass TD tube and desorbed at the rate of 60 ml min^-1^ for 8 min at 200°C under a stream of N_2_ gas. The desorbed substances were cryo-focused at -60°C. After that the trap was heated to 230°C, and the analytes were injected using splitless mode on to a Rtx-5MS GC column (thickness- 0.25 µm, length- 30 m, diam-0.25 µm) using helium as carrier gas. The oven temperature was programmed as follows: 45°C initial (held for 2 min), then ramped to 250°C at a rate of 6°/min, and finally to 300°C (held for 3 min). The run time was approximately 40 min for each sample. Compounds in Table 2 were identified by comparison of their mass spectra and retention times to those of authentic standards, expect for 2,3-dihydrobenzofuran and methyl (*E*)-cinnamate whose identity was based only on comparison of their mass spectra to those found in reference data bases.

Kallenbach M, Oh Y, Eilers EJ, Veit D, Baldwin IT, Schuman MC (2014) A robust, simple, high-throughput technique for time-resolved plant volatile analysis in field experiments. Plant J 78:1060-1072
